# Supplementary material for: Critical role of interferons in gastrointestinal injury repair
Source: Nat Commun. 2021 May 11;12:2624. doi: 10.1038/s41467-021-22928-0 (PMC8113246; doi:10.1038/s41467-021-22928-0)
Supplement: Supplementary file 1 — Supplementary Information [file 41467_2021_22928_MOESM1_ESM.pdf]

Supplementary Information

Critical Role of Interferons in Gastrointestinal Injury Repair

Constance McElrath, *et al.*, Sergei V Kotenko*

*Corresponding authors. Email: kotenkse@njms.rutgers.edu

Supplementary Figure 1

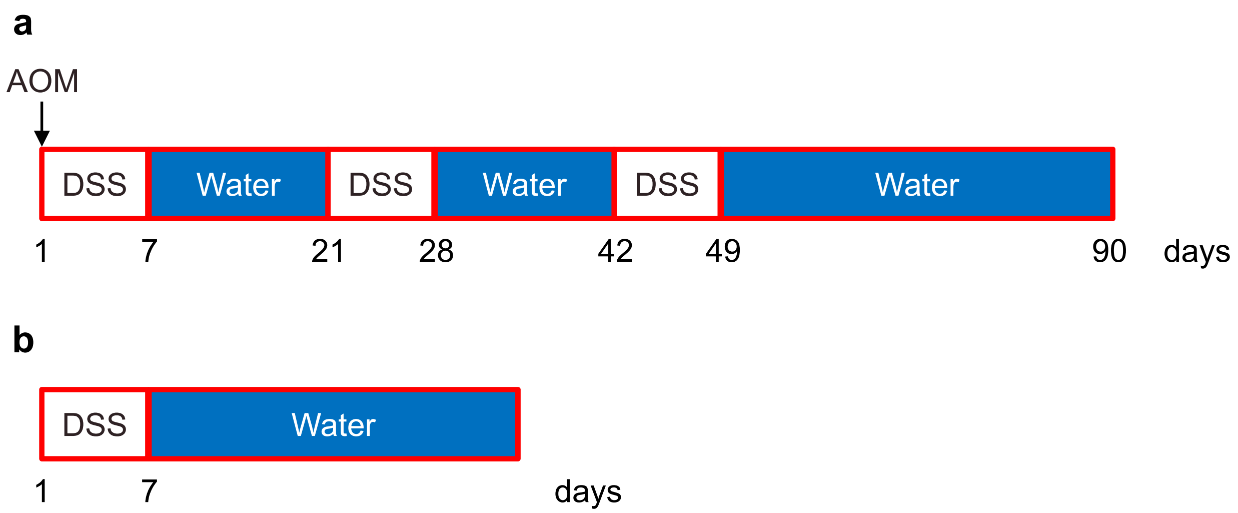

Supplementary Figure 1. AOM/DSS and DSS models. Timelines of (**a**) the AOM/DSS model of inflammation-induced colorectal cancer and (**b**) the DSS model of ulcerative colitis are schematically shown.

Supplementary Figure 2

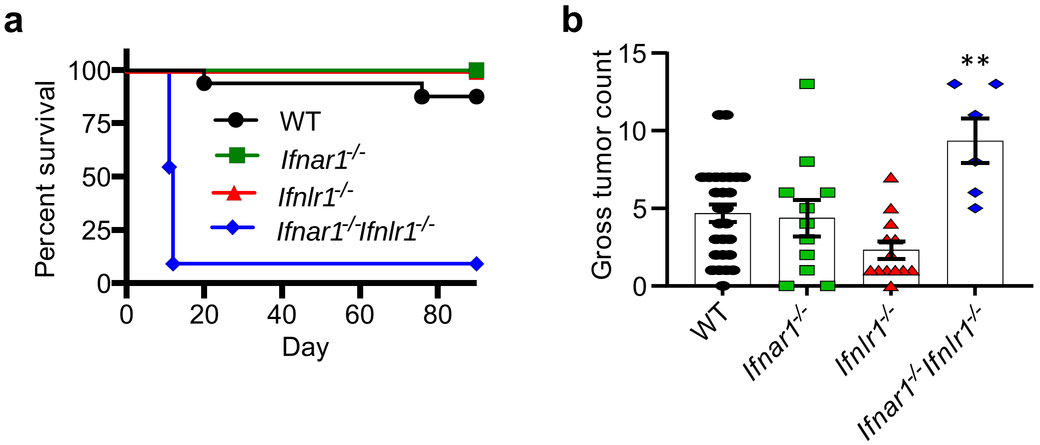

Supplementary Figure 2. Enhanced susceptibility of *Ifnar1$^{-/-}$Ifnlr1$^{-/-}$* mice to AOM/DSS treatment. a, b 6-8 week old wild type (WT), single IFNR-deficient *Ifnar1$^{-/-}$* or *Ifnlr1$^{-/-}$* mice or double IFNR-deficient *Ifnar1$^{-/-}$Ifnlr1$^{-/-}$* mice were given a single IP injection of the carcinogen AOM, followed by three cycles of 7 day 1.5% DSS in drinking water, followed by 14 day of regular drinking water. Mice were monitored daily for (**a**) survival (WT, n=16; *Ifnar1$^{-/-}$*, n=15; *Ifnlr1$^{-/-}$*, n=16; *Ifnar1$^{-/-}$Ifnlr1$^{-/-}$*, n=22). (**b**) Tumor burden was quantitated on day 90 (WT, n=27; *Ifnar1$^{-/-}$*, n=11; *Ifnlr1$^{-/-}$*, n=13; *Ifnar1$^{-/-}$Ifnlr1$^{-/-}$*, n=6). Data are pooled from more than two independent experiments. Symbols represent values of individual mice (**b**). Quantitative data were analyzed using one-way ANOVA followed by Bonferroni's multiple comparisons test and represent mean values with SEM. ***P*=0.0039 and is for the indicated animal group compared with control group of WT mice.

Supplementary Figure 3

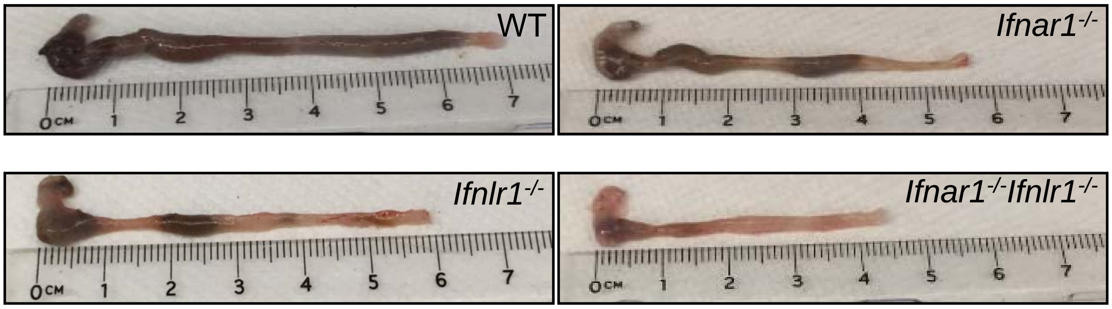

Supplementary Figure 3. Effects of DSS treatment on the size of large intestines. Representative macroscopic images of the large intestines from mice sacrificed on day 11 following treatment with 1.5% DSS for 7 days (n=10). Data are representative of two independent experiments.

Supplementary Figure 4

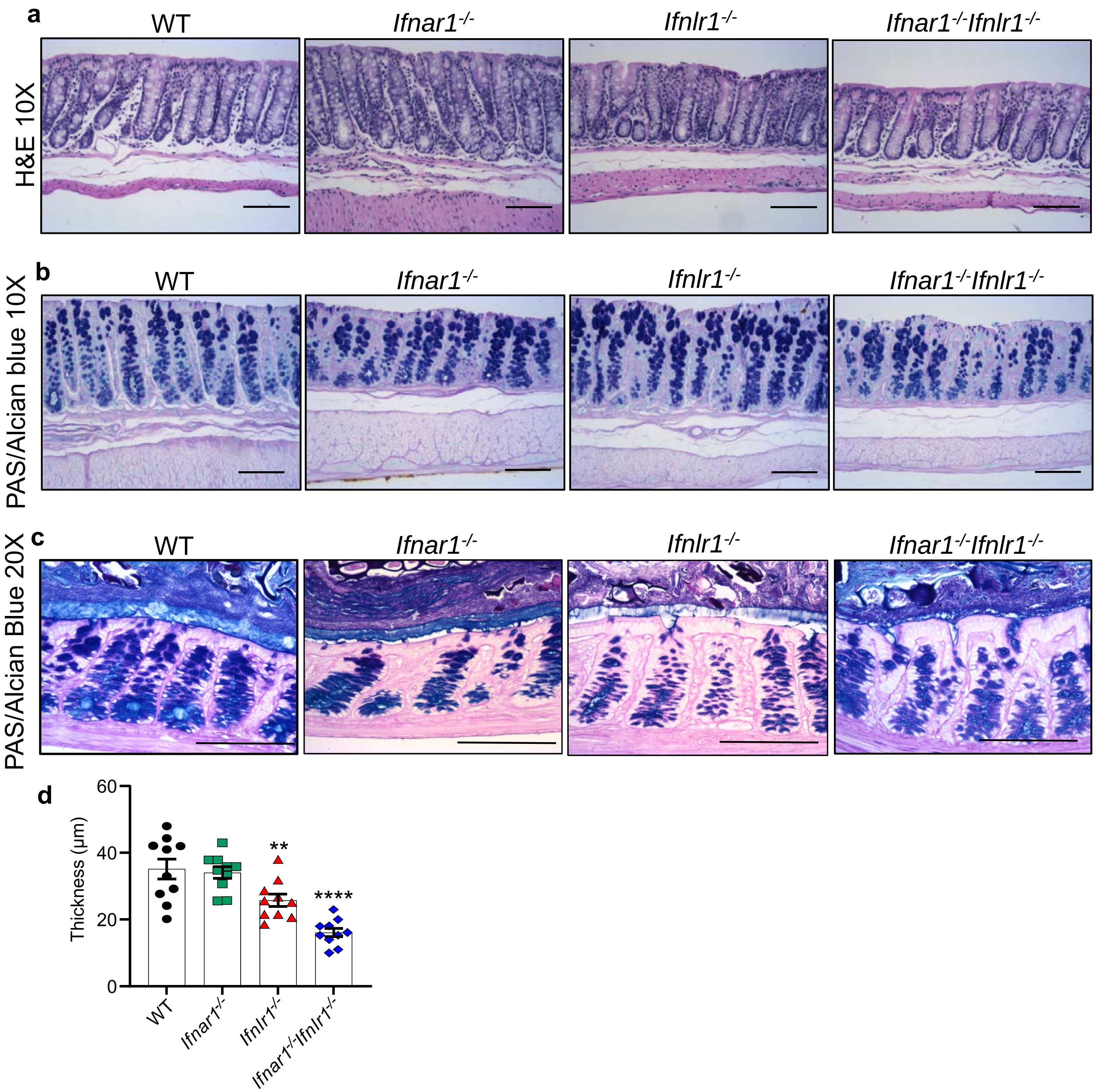

Supplementary Figure 4. Histological evaluation of the colons of WT and IFNR-deficient mice during homeostasis. a-d 6-8 week old WT and various IFNR-deficient mice that did not receive the DSS solution (naïve mice; n=5) were euthanized and colon histology was analyzed on FFPE tissue slides by (**a**) H&E and (*B*) PAS/Alcian Blue staining. Representative images are shown. **c, d** Colon tissue was also fixed in Carnoy's solution, paraffin-embedded and tissue slides were stained with PAS/Alcian Blue. **c** Representative images of inner mucus layer with (**d**) quantified data of the mucus layer thickness are shown. Symbols represent individual measurements of the inner mucus layer thickness (2 measurements/slide/animal; n=5). Scale bars are 100 μM. Data are representative of two independent experiments. Quantitative data were analyzed using one-way ANOVA followed by Bonferroni's multiple comparisons test and present mean values with SEM. $^{**}P=0.0084$, $^{****}P\leq0.0001$. *P* values are for the indicated animal group compared with control group of WT mice

Supplementary Figure 5

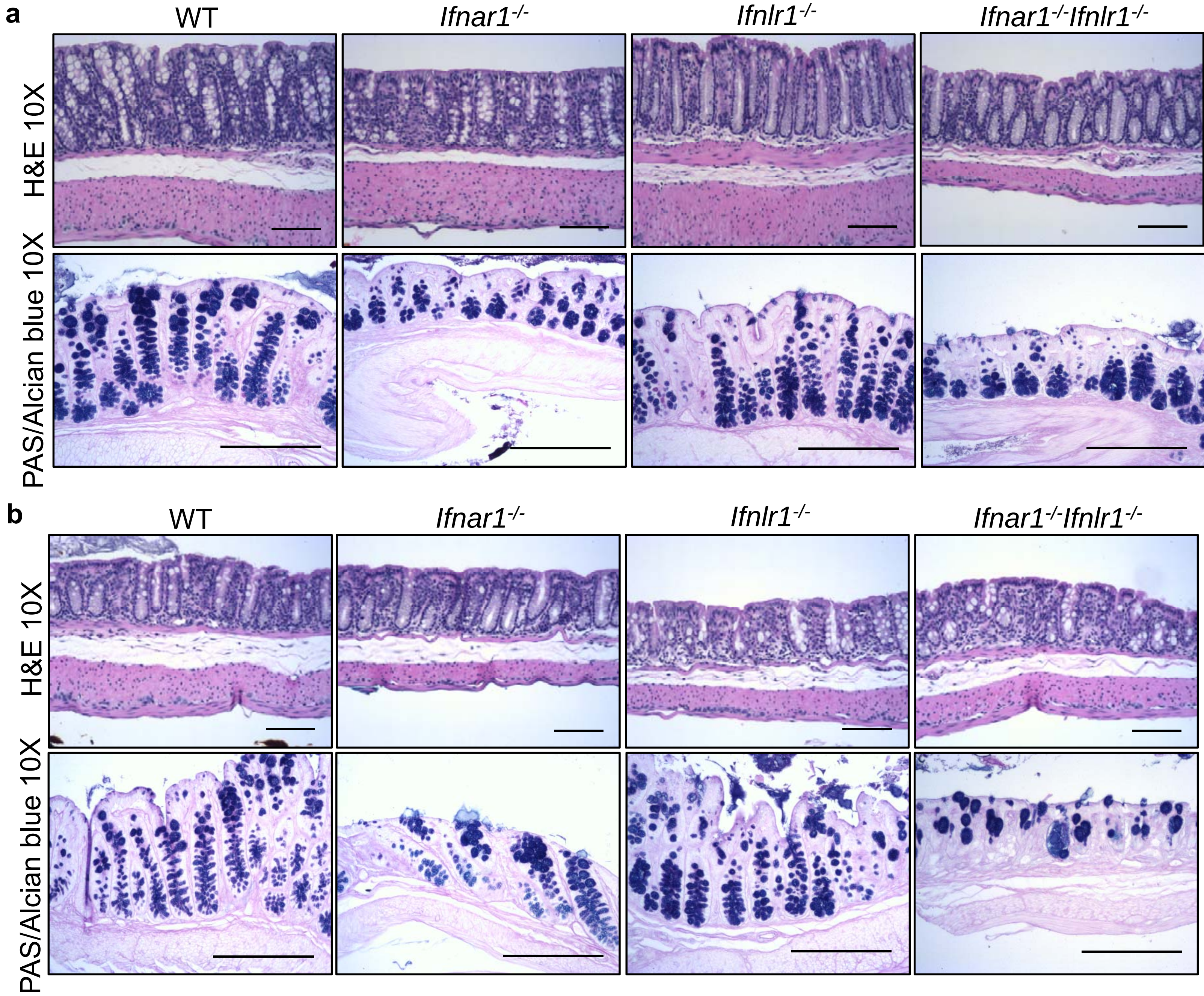

Supplementary Figure 5. Histological changes in colons of WT and IFNR-deficient mice on days 5 and 8 of DSS-induced colitis. a, b 6-8 week old WT and various IFNR-deficient mice (n=5) were treated with 1.5% DSS in drinking water for 7 days followed with regular water for recovery, and euthanized on day 5 or 8. Representative H&E and PAS/Alcian Blue staining of colons of mice on (**a**) day 5 and (**b**) day 8 of DSS-induced colitis are shown. Scale bars are 100 μM. Data are representative of two independent experiments.

Supplementary Figure 6

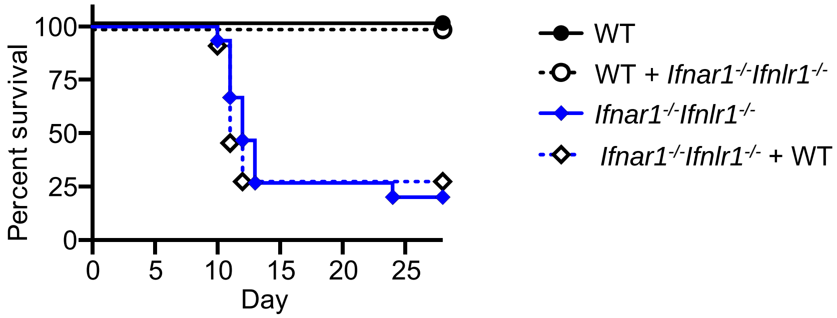

Supplementary Figure 6. Effects of co-housing of WT and *Ifnar1*$^{-/-}$*Ifnlr1*$^{-/-}$ mice on their sensitivity to DSS treatment. 4 week old WT and *Ifnar1*$^{-/-}$*Ifnlr1*$^{-/-}$ mice (WT, n=5; WT+*Ifnar1*$^{-/-}$*Ifnlr1*$^{-/-}$, WT n=10; *Ifnar1*$^{-/-}$*Ifnlr1*$^{-/-}$+WT, *Ifnar1*$^{-/-}$*Ifnlr1*$^{-/-}$ n=11; *Ifnar1*$^{-/-}$*Ifnlr1*$^{-/-}$, n=15) were co-housed for a minimum of 3 weeks, subsequently treated with 1.5% DSS in drinking water for 7 days, followed by regular water, and monitored for survival. Data are pooled from two independent experiments.

Supplementary Figure 7

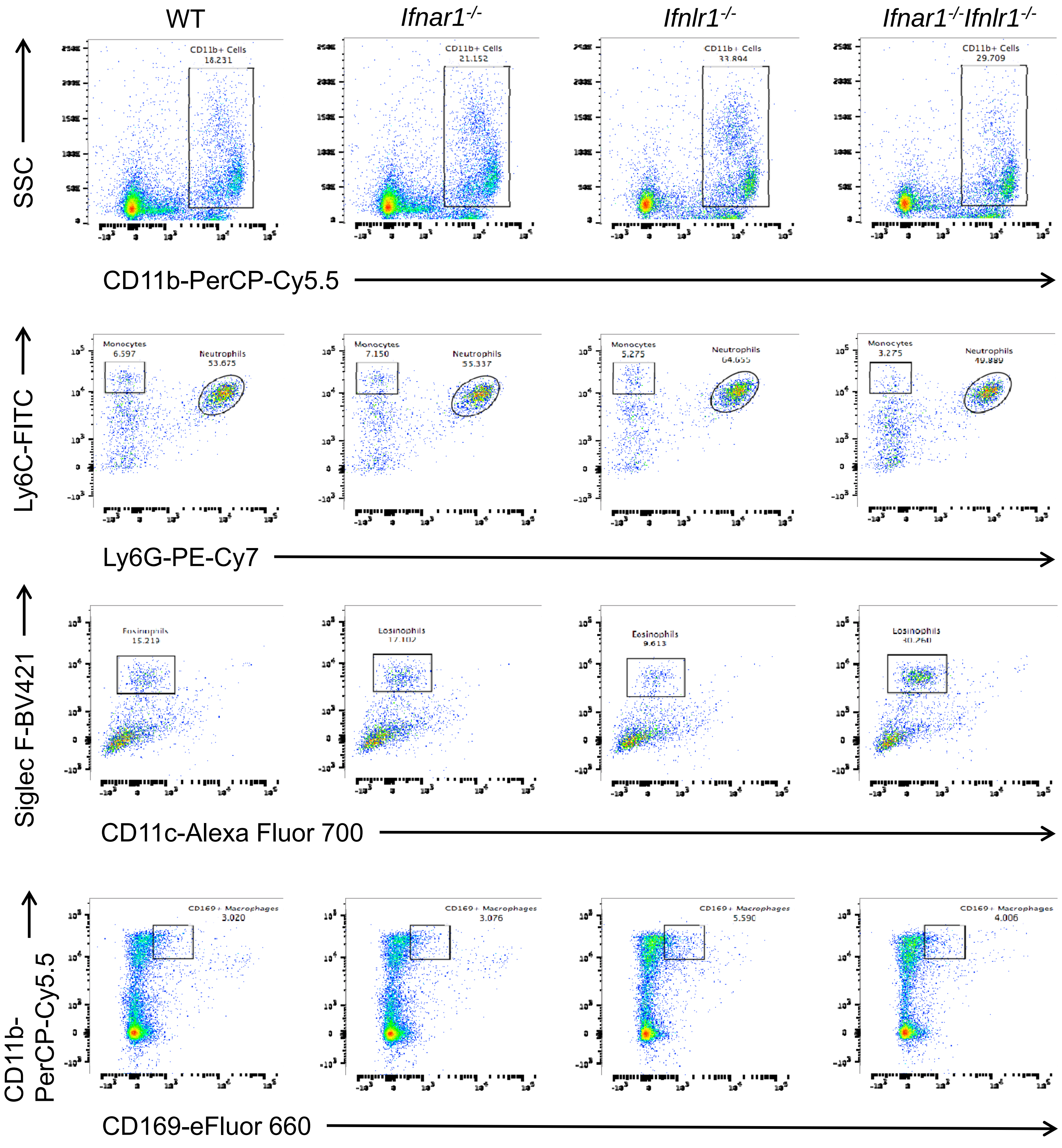

Supplementary Figure 7. Gating strategy to enumerate colon immune cell infiltrates. 6-8 week old WT and various IFNR-deficient mice were treated with 1.5% DSS for 7 days, followed by regular drinking water. On day 8, colon immune cell infiltrates were quantitated by flow cytometry (n=5-6). Cells were gated on live, $CD45^+$ cells, then $CD11b^+Ly6G^+$ for neutrophils, $CD11b^+Ly6C^+$ for monocytes, $CD11b^+CD11c^-Siglec\ F^+$ for eosinophils, and $CD11b^+CD169^+$ for macrophages. Representative data are shown.

Supplementary Figure 8

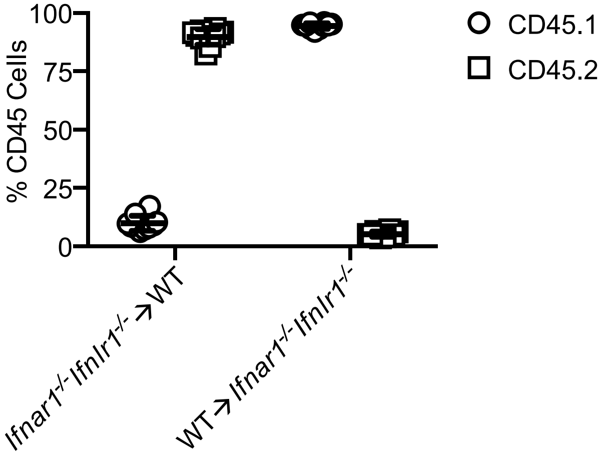

Supplementary Figure 8. Percent engraftment in bone marrow chimera mice. Following lethal irradiation and adoptive transfer of donor bone marrow cells, mice were given 6 weeks for reconstitution of bone marrow niche. Percent donor and recipient cells in peripheral blood was evaluated by flow cytometry for CD45.1$^+$ and CD45.2$^+$ cells (n=10). Quantitative data are presented as mean values with SEM.

Supplementary Figure 9

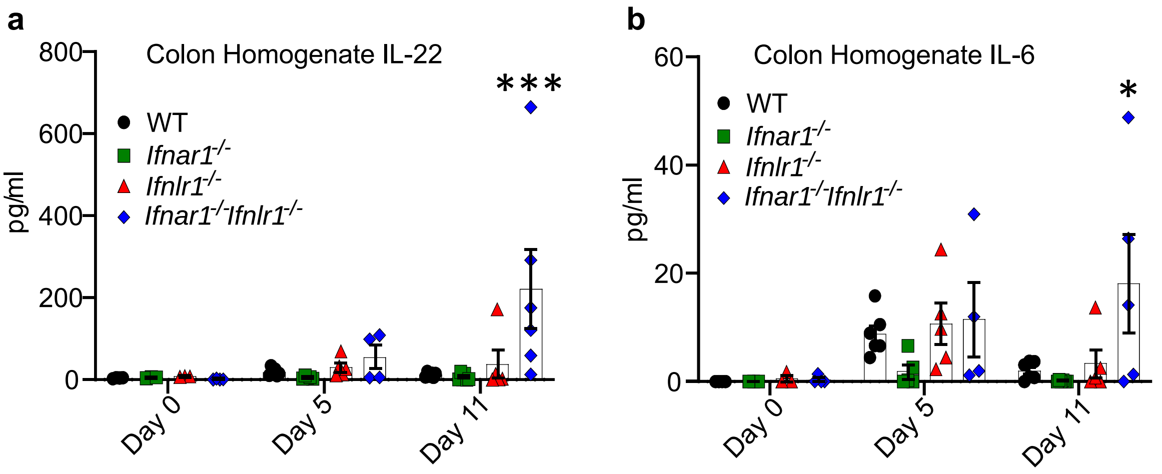

Supplementary Figure 9. Levels of colonic IL-22 and IL-6 expression in WT and IFNR-deficient mice following DSS treatment. a, b 6-8 week old WT, single or double IFNR-deficient mice were exposed to 1.5% DSS in drinking water for 7 days followed by regular drinking water. Mice were euthanized on selected days and levels of colonic (**a**) IL-22 and (**b**) IL-6 were quantitated by MSD ELISA (**a** WT, n=6; *Ifnar1*$^{-/-}$, n=6; *Ifnlr1*$^{-/-}$, n=5; *Ifnar1*$^{-/-}$*Ifnlr1*$^{-/-}$, n=6; **b** WT, n=6; *Ifnar1*$^{-/-}$, n=6; *Ifnlr1*$^{-/-}$, n=5; *Ifnar1*$^{-/-}$*Ifnlr1*$^{-/-}$, n=5). Symbols represent values of individual mice. Quantitative data were analyzed using two-way ANOVA followed by Bonferroni's multiple comparisons test and represent mean values with SEM. **a** ****P*=0.0006. **b** **P*=0.0102. *P* values are for the indicated animal group compared with control group of WT mice.

Supplementary Figure 10

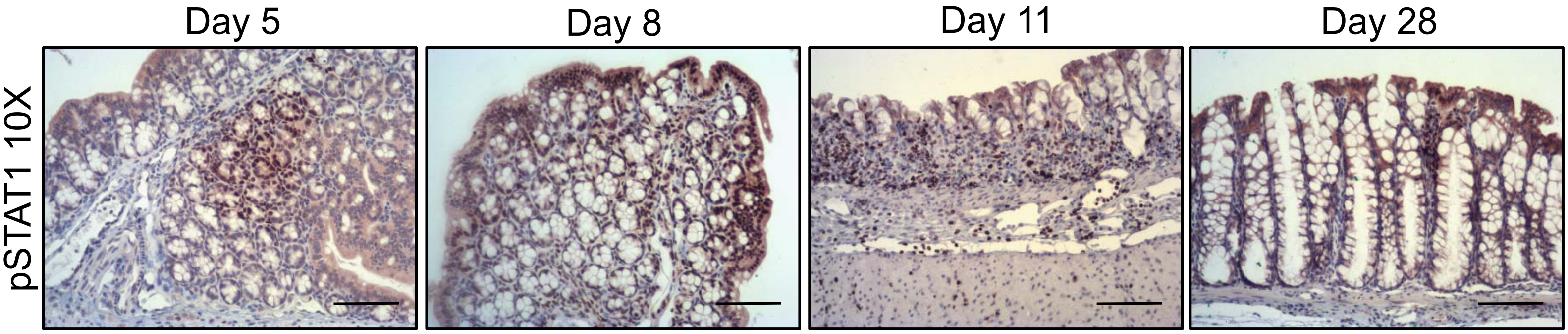

Supplementary Figure 10. STAT1 phosphorylation in colon during various phases of DSS-induced colitis. WT mice were exposed to 1.5% DSS in drinking water for 7 days, followed by regular drinking water. Mice were euthanized on select days and STAT1 phosphorylation was evaluated in the colon by IHC staining with antibodies against phosphorylated STAT1 (n=5). Representative images are shown. Scale bars are 100 μM.

Supplementary Figure 11

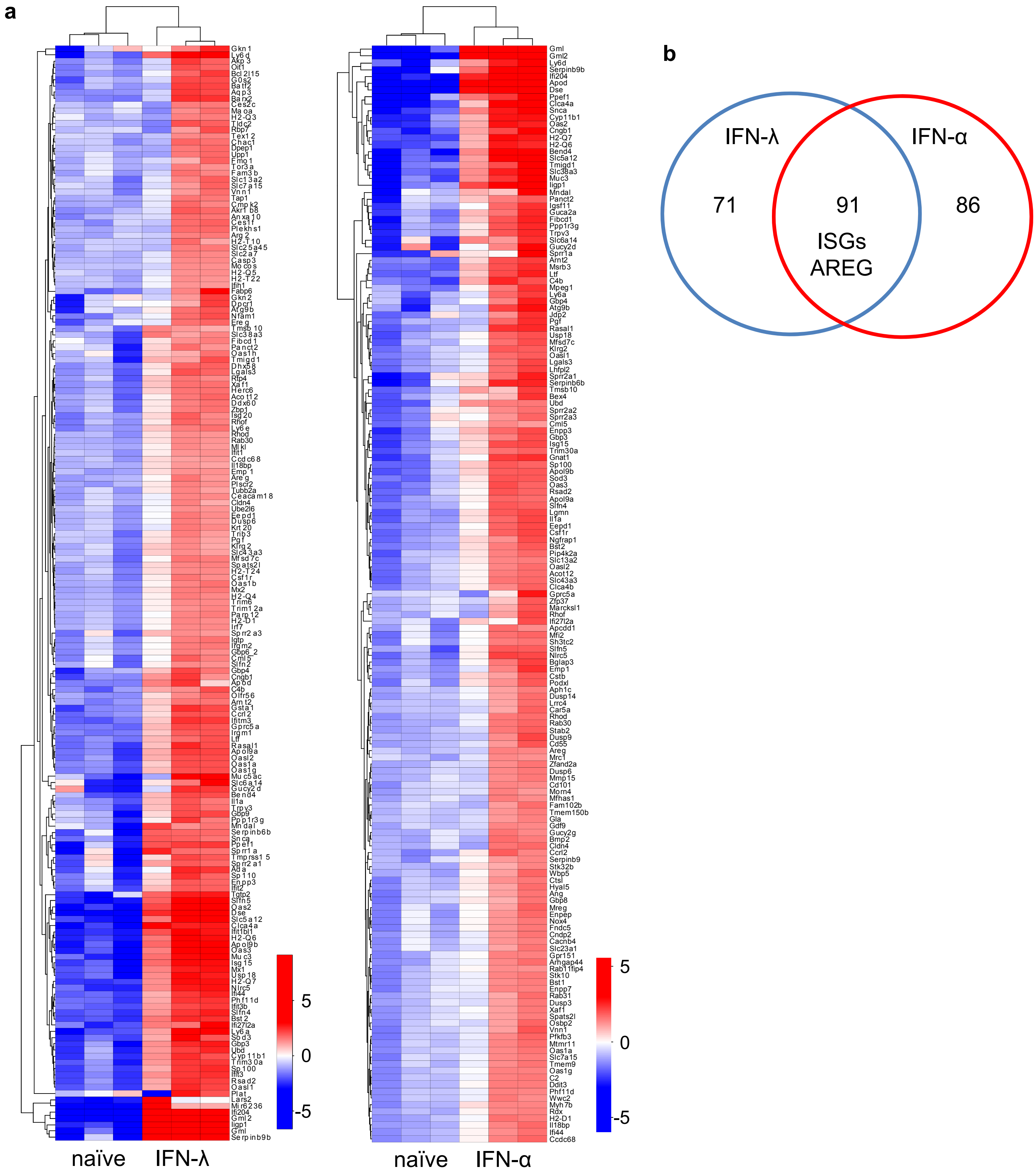

Supplementary Figure 11. Schematic presentation of RNAseq data analyses for the genes differentially expressed in *in vivo* treated epithelial cells. a, b 6-8 day old WT mice were treated daily with mIFN-α or mIFN-λ for 3 days and intestinal epithelial cells were FAC-sorted and subjected to RNAseq (n=3). **a** Hierarchical clustering heat maps show sets of genes significantly up-regulated more than 5 fold (FC > 5; the significance was determined by setting false discovery rate (FDR) < 0.05) in response to either mIFN-α or mIFN-λ. Color intensity indicates the level of gene expression (red for up-regulation and blue for down-regulation). **b** Venn diagram summarizes overlap between these sets of genes and reveals the up-regulation of AREG transcripts by both types of IFNs.

Supplementary Figure 12

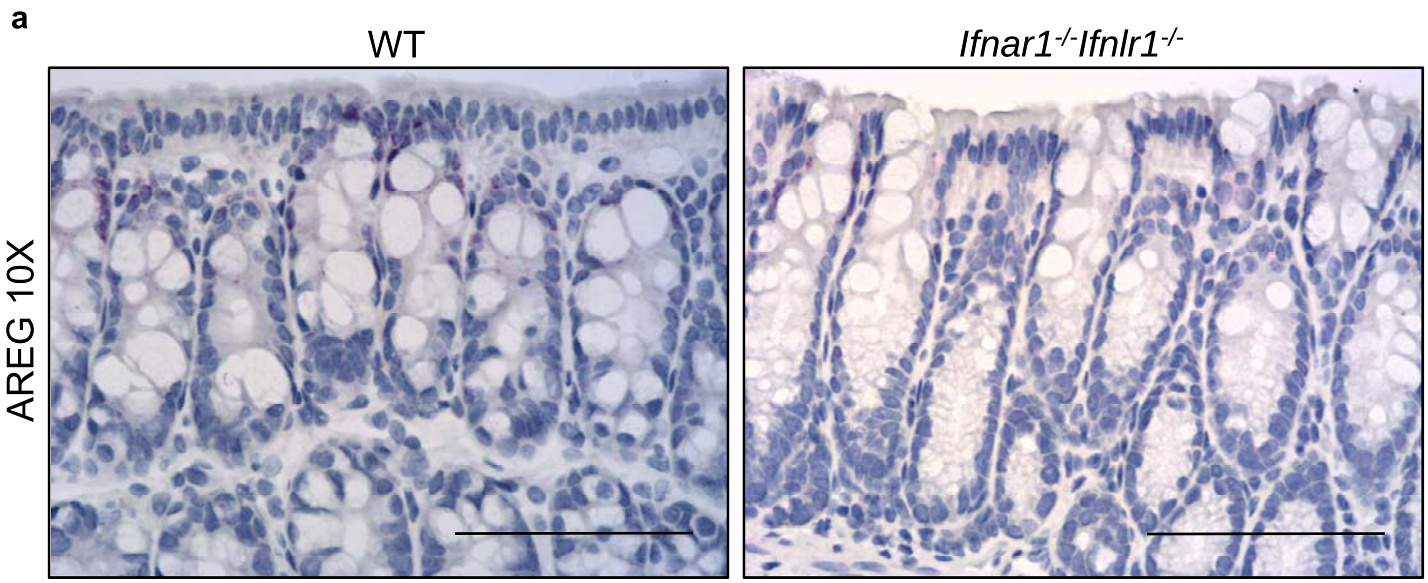

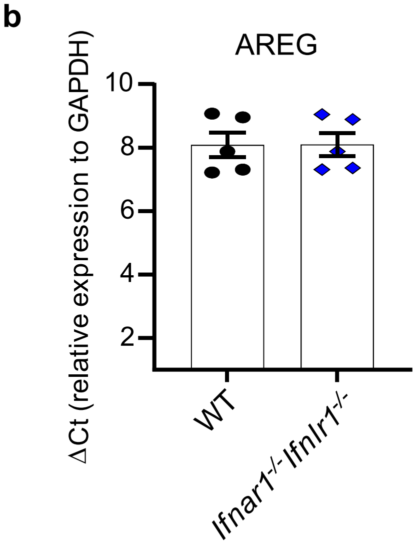

Supplementary Figure 12. AREG transcript expression in colons of WT and *Ifnar1$^{-/-}$Ifnlr1$^{-/-}$* mice during homeostasis. a, b 6-8 week old, WT and *Ifnar1$^{-/-}$Ifnlr1$^{-/-}$* mice that did not receive the DSS solution (naïve mice) were euthanized and the levels of AREG transcript expression were evaluated by (**a**) *in situ* hybridization in colon tissue slides (n=5) and (**b**) qPCR in colon homogenates (n=5). Scale bars are 100 µM. Symbols represent values of individual mice. Quantitative data represent mean values with SEM.

Supplementary Figure 13

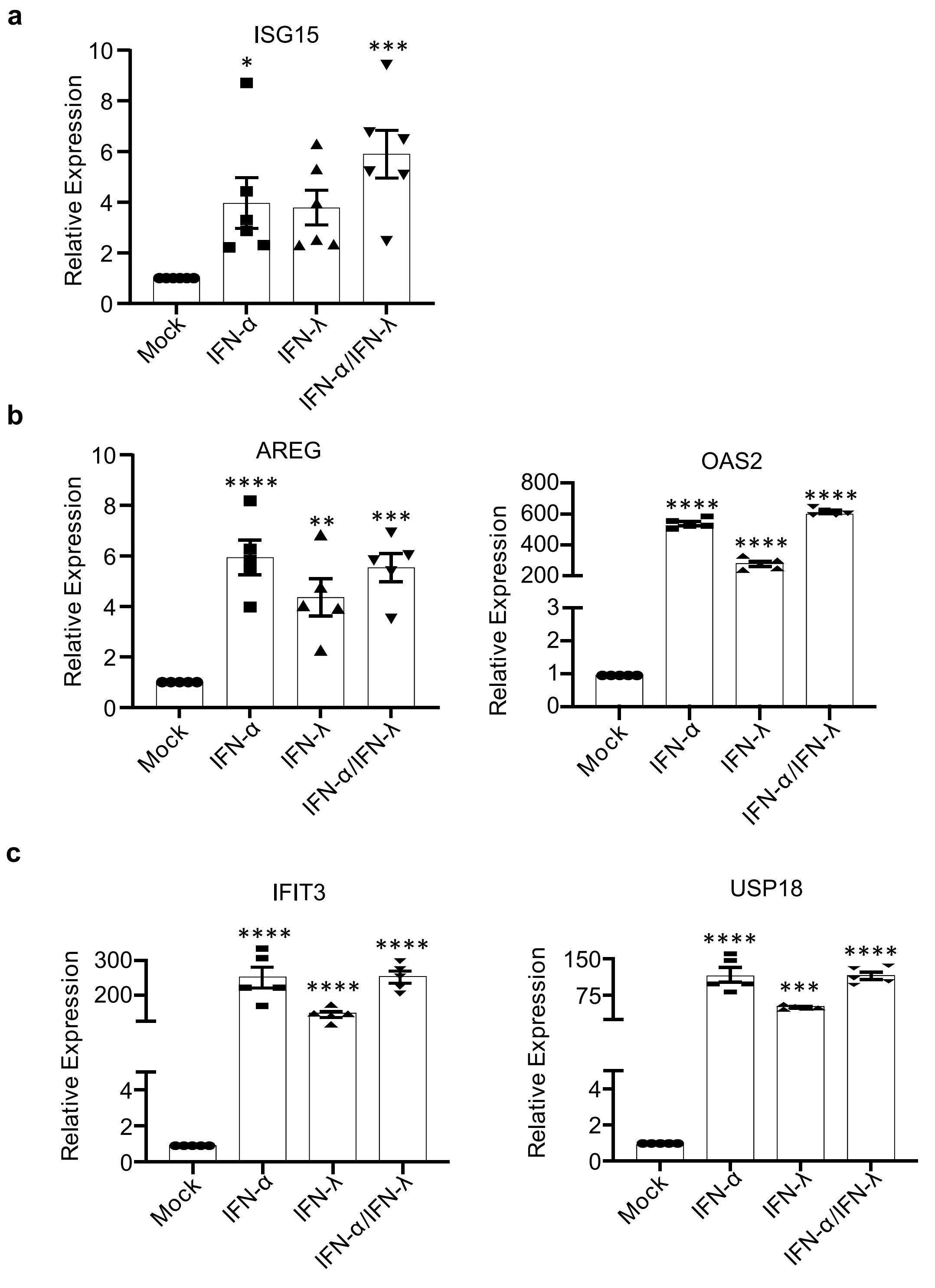

Supplementary Figure 13. Effects of IFN treatment on the levels of AREG and classical ISG transcription. **a** Murine intestinal epithelial cells (mIECs), **b** murine lung epithelial cells (MLE-15), or **c** FAC-sorted CD45^{+} cells from bone marrow of WT mice were left untreated or treated with IFNs for 3 h and expression levels of AREG and indicated ISG transcripts were evaluated by qPCR (**a** n=6; **b** n=5; **c** n=5). Data are pooled from two independent experiments. Symbols represent individual measurements. Quantitative data were analyzed using one-way ANOVA followed by Bonferroni's multiple comparisons test and represent mean values with SEM. **a** *P=0.0389 (IFN-α), ***P=0.0007 (IFN-α/IFN-λ). **b** **P=0.0024 (IFN-λ), ***P=0.0001 (IFN-α/IFN-λ). **c** ***P=0.0008 (IFN-λ). ****P≤0.0001. P values are for the indicated treatment compared with mock.

Supplementary Figure 14

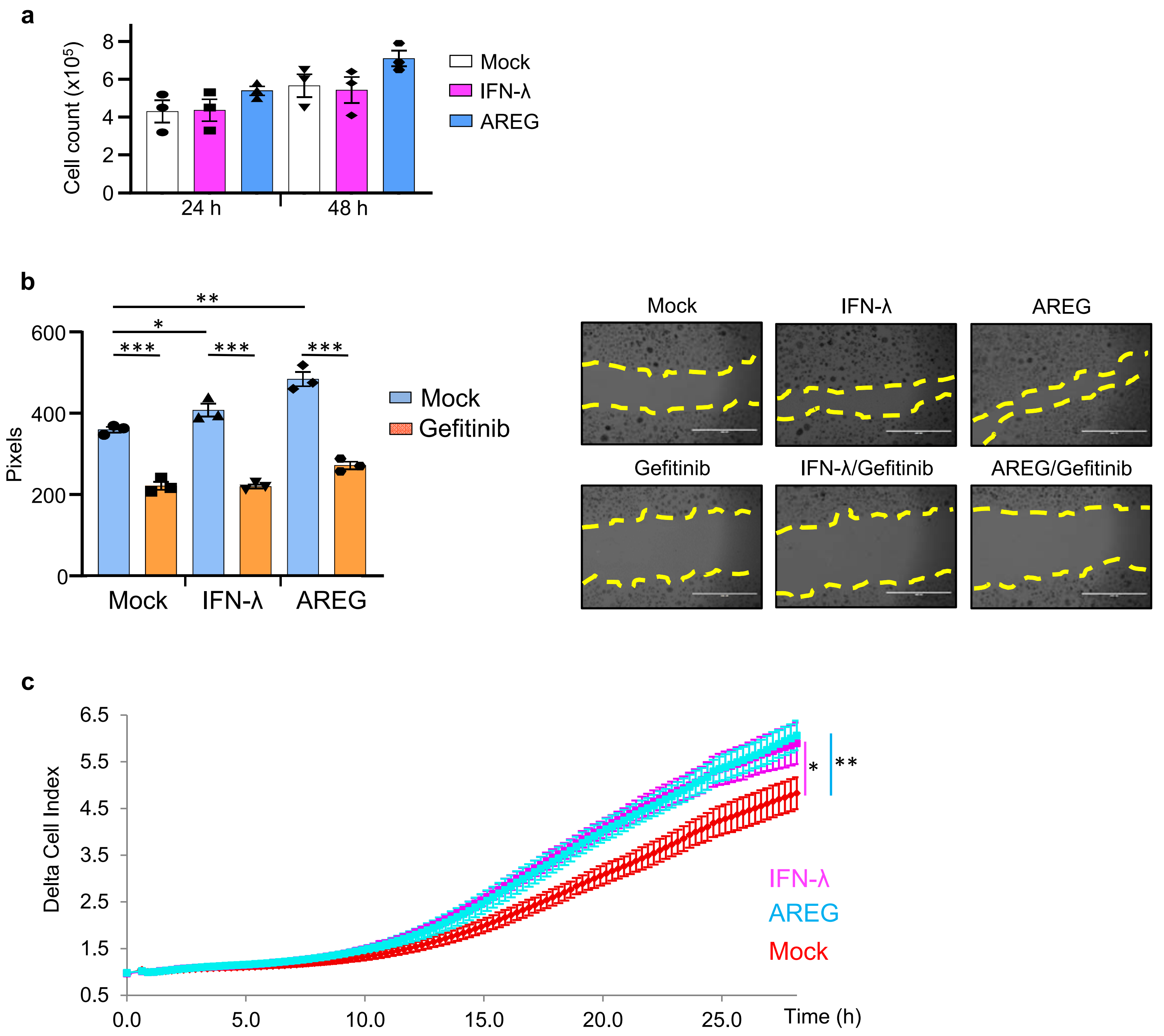

Supplementary Figure 14. Effects of IFN-λ and AREG on cell proliferation and migration. a mIECs were left untreated or treated with IFN-λ2 or AREG and cell numbers were counted at 24 and 48 h (n=3 done in triplicates per condition). **b** Confluent monolayer of mIECs was scratched and cell migration into the gap was assessed at 24 h for the untreated cells or cells treated with IFN-λ2 or AREG with or without gefitinib and presented as the distance migrated by the cells form the edge of the gap in 24 h measured in pixels (n=3 done in triplicates per condition). Representative images at 24 h are shown. **c** mIECs were seeded in the upper chamber of the XCELLigence RTCA DP CIM-16 plate and changes in cell index (CI) depicting cellular migration to the bottom chamber were assessed every 10 min for 28 h for the untreated cells or cells treated with pegylated IFN-λ2 or AREG (n=3, data are representative of two independent experiments). **a, b** Symbols represent individual measurements. Quantitative data were analyzed using (**a, b**) pairwise two-tailed unpaired *t*-test or (**c**) one-way ANOVA followed by Tukey's multiple comparisons test. **b** **P*=0.0459 (mock versus IFN-λ), ***P*=0.0026 (mock versus AREG), ****P*=0.0003 (mock versus mock+gefitinib) ****P*=0.0003 (IFN-λ versus IFN-λ+gefitinib), ****P*=0.0004 (AREG versus AREG+gefitinib). **c** **P*=0.0383 (mock versus IFN-λ), ***P*=0.0095 (mock versus AREG).
