# Supplementary material for: Critical role of interferons in gastrointestinal injury repair
Source: Nat Commun. 2021 May 11;12:2624. doi: 10.1038/s41467-021-22928-0 (PMC8113246; doi:10.1038/s41467-021-22928-0)
Supplement: Supplementary file 3 — Reporting Summary [file 41467_2021_22928_MOESM3_ESM.pdf]

nature research

Corresponding author(s): Sergei V Kotenko

Last updated by author(s): Mar 30, 2021

Reporting Summary

Nature Research wishes to improve the reproducibility of the work that we publish. This form provides structure for consistency and transparency in reporting. For further information on Nature Research policies, see Authors & Referees and the Editorial Policy Checklist .

Statistics

For all statistical analyses, confirm that the following items are present in the figure legend, table legend, main text, or Methods section.

n/a	Confirmed	
☐	☒	The exact sample size (n) for each experimental group/condition, given as a discrete number and unit of measurement
☐	☒	A statement on whether measurements were taken from distinct samples or whether the same sample was measured repeatedly
☐	☒	The statistical test(s) used AND whether they are one- or two-sided<br>*Only common tests should be described solely by name; describe more complex techniques in the Methods section.*
☒	☐	A description of all covariates tested
☐	☒	A description of any assumptions or corrections, such as tests of normality and adjustment for multiple comparisons
☐	☒	A full description of the statistical parameters including central tendency (e.g. means) or other basic estimates (e.g. regression coefficient) AND variation (e.g. standard deviation) or associated estimates of uncertainty (e.g. confidence intervals)
☐	☒	For null hypothesis testing, the test statistic (e.g. F, t, r) with confidence intervals, effect sizes, degrees of freedom and P value noted<br>*Give P values as exact values whenever suitable.*
☒	☐	For Bayesian analysis, information on the choice of priors and Markov chain Monte Carlo settings
☒	☐	For hierarchical and complex designs, identification of the appropriate level for tests and full reporting of outcomes
☒	☐	Estimates of effect sizes (e.g. Cohen's d, Pearson's r), indicating how they were calculated

Our web collection on statistics for biologists contains articles on many of the points above.

Software and code

Policy information about availability of computer code

Data collection	LSRFortessa X-20 with FACSDiva v.8.0 (BD Biosciences), NextSeq 500 (Illumina), CFX96 RT PCR Detection System (Bio-Rad), MS2400 imager (Meso Scale Diagnostics), in vivo imaging system (IVIS 200, Xenogen), Vi-Cell cell analyzer (Beckman Coulter), Bioanalyzer 2100 and XCELLigence RTCA DP instrument (Agilent)
Data analysis	FlowJo v.10.2, GraphPad Prism v.9.0.0, ImageJ 1.x, FastX clipper (FASTX-Toolkit/0.0.14), Package HTSeq-0.6.1, Trimmomatic-0.33, Tophat v.2.0.13, Bioconductor package edgeR v.3.8.6 with limma 3.22.7

For manuscripts utilizing custom algorithms or software that are central to the research but not yet described in published literature, software must be made available to editors/reviewers. We strongly encourage code deposition in a community repository (e.g. GitHub). See the Nature Research guidelines for submitting code & software for further information.

Data

Policy information about availability of data

All manuscripts must include a data availability statement. This statement should provide the following information, where applicable:
- Accession codes, unique identifiers, or web links for publicly available datasets
- A list of figures that have associated raw data
- A description of any restrictions on data availability

RNA-seq data reported in this study have been deposited in the NCBI SRA with the accession number PRJNA579563. Other data supporting the findings of this study are available from the corresponding author upon reasonable request.

Field-specific reporting

Please select the one below that is the best fit for your research. If you are not sure, read the appropriate sections before making your selection.

☒ Life sciences ☐ Behavioural & social sciences ☐ Ecological, evolutionary & environmental sciences

For a reference copy of the document with all sections, see nature.com/documents/nr-reporting-summary-flat.pdf

Life sciences study design

All studies must disclose on these points even when the disclosure is negative.

Sample size

For animal experiments, several pilot studies were initially conducted to evaluate phenotypes, since no information on the experimental outcome was available when the studies were designed. These pilot experiments showed high reproducibility of the results and low variability within each mouse strain, and demonstrated that 5-7 animals per group were sufficient to provide statistically significant data. Therefore, a minimum of 5 mice per group/treatment were used with at least 2 replications. However, larger numbers of Ifnar1-/-Ifnlr1-/- mice were typically used for experiments (Fig. 1 and Fig. S2) to account for their enhanced susceptibility and ensure that sufficient number of animals would survive to the experimental endpoint.

For experiments conducted with cell lines, no sample size calculation was performed. Biological triplicate samples were used in experiments as this is a standard practice in the field.

Data exclusions

No data were excluded from the analysis.

Replication

Experiments were conducted at least 2 independent times, resulting in consistent and reproducible results with statistical significance.

Randomization

Typically experiments were conducted with mice from 2-3 litters per strain, and animals of each strain were randomly selected into the DSS-treated group to normalize for the equal number of males and females, age and initial weight across the strains to remove age-, gender- and cage-, litter-related bias in the results. For rescue experiments, mice were randomly assigned to AREG and PBS treatments. All animals were bred and kept in the same animal facility to have similar baseline immune and homeostatic conditions.

Blinding

Experiments were not conducted blindly since cages with DDS-containing water needed to be specifically labeled per IACUC requirements. In addition, investigators were not blinded to genotypes or treatment groups since the change of body weight is a non-subjective measurement. However, pathology slides and other tissue specimens were analyzed blindly. Investigators were not blinded to group allocation for other experiments because treatments, data collection and analysis were done by the same people and non-subjective measurements (i.e. colon weight and length; flow cytometry, ELISA and qPCR data; luminescence flux, tumor counts and cell migration distance) were collected and analyzed.

Reporting for specific materials, systems and methods

We require information from authors about some types of materials, experimental systems and methods used in many studies. Here, indicate whether each material, system or method listed is relevant to your study. If you are not sure if a list item applies to your research, read the appropriate section before selecting a response.

Materials & experimental systems

n/a	Involved in the study	
☐	☒	Antibodies
☐	☒	Eukaryotic cell lines
☒	☐	Palaeontology
☐	☒	Animals and other organisms
☒	☐	Human research participants
☒	☐	Clinical data

Methods

n/a	Involved in the study	
☒	☐	ChIP-seq
☐	☒	Flow cytometry
☒	☐	MRI-based neuroimaging

Antibodies

Antibodies used

The following antibodies were used:

i) for flow cytometry: Ly6C (#561085, clone AL-21), CD11b (#561114, clone M1/70), Siglec F (#565934, clone E50-2440), CD11c (#560583, clone HL3), EpCAM (#563477, clone G8.8), Ly6G (#560600, clone 1A8), MHCII (#563414, clone M5/11.415.2), CD45.1 (#553775, clone A20), CD45.2 (#564279, clone 30-F11) (all from BD); CD169 (#50-5755-82, clone SER-4) (eBioscience); and F4/80 (#123117, clone BM8) (BioLegend);

ii) for immunohistochemistry: rabbit monoclonal Ki-67 (RM91106, clone SP6) (Fisher Scientific) and phospho-STAT1 (#9167R, clone 58D6) (Cell Signaling), and UltraTek anti-rabbit biotinylated (ABK, ready-to-use) (ScyTek).

Validation

Antibodies were validated for their use by the respective manufacturers:

-Ly6C: https://www.bdbiosciences.com/us/reagents/research/antibodies-buffers/immunology-reagents/anti-mouse-antibodies/

cell-surface-antigens/fitc-rat-anti-mouse-ly-6c-al-21/p/553104
-CD11b, https://www.bdbiosciences.com/us/applications/research/stem-cell-research/mesenchymal-stem-cell-markers-bone-marrow/mouse/negative-markers/percp-cy55-rat-anti-cd11b-m170/p/561114
-Siglec F: https://www.bdbiosciences.com/us/applications/research/b-cell-research/surface-markers/mouse/bv421-rat-anti-mouse-siglec-f-e50-2440/p/562681
-CD11c: https://www.bdbiosciences.com/us/reagents/research/antibodies-buffers/immunology-reagents/anti-mouse-antibodies/cell-surface-antigens/alexa-fluor-700-hamster-anti-mouse-cd11c-hl3/p/560583
-EpCAM: https://www.bdbiosciences.com/us/applications/research/stem-cell-research/cancer-research/mouse/pe-rat-anti-mouse-cd326-g88/p/563477
-Ly6G: https://www.bdbiosciences.com/us/reagents/research/antibodies-buffers/immunology-reagents/anti-mouse-antibodies/cell-surface-antigens/apc-cy7-rat-anti-mouse-ly-6g-1a8/p/560600
-MHCII: https://www.bdbiosciences.com/us/reagents/research/antibodies-buffers/immunology-reagents/anti-mouse-antibodies/cell-surface-antigens/bv711-rat-anti-mouse-i-ai-e-m5114152-also-known-as-m5114/p/563414
-CD45.1: https://www.bdbiosciences.com/us/applications/research/stem-cell-research/cancer-research/mouse/fitc-mouse-anti-mouse-cd451-a20/p/553775
-CD45.2: https://www.bdbiosciences.com/us/reagents/research/antibodies-buffers/immunology-reagents/anti-mouse-antibodies/cell-surface-antigens/buv395-rat-anti-mouse-cd45-30-f11/p/564279
-CD169: https://www.thermofisher.com/antibody/product/CD169-Siglec-1-Antibody-clone-SER-4-Monoclonal/50-5755-82
-F4/80: https://www.biolegend.com/en-us/products/apc-cyanine7-anti-mouse-f4-80-antibody-4072
-pSTAT1: https://www.cellsignal.com/products/primary-antibodies/phospho-stat1-tyr701-58d6-rabbit-mab/9167
-Ki-67: https://assets.fishersci.com/TFS-Assets/APD/Specification-Sheets/D12537~.pdf
-ant-rabbit biotinylated: https://www.scytek.com/products/201.78-ABK125-UltraTek-Anti-Rabbit.asp

Eukaryotic cell lines

Policy information about cell lines

Cell line source(s)	Immortalized murine intestinal epithelial cells (mIEC-1) were obtained from Tobias May where this cell line was created. A clonal population (clone G7) was selected in the lab based on its high trans-epithelial electrical resistance (TEER > 3,000Ω/square cm) when grown as polarized culture in trans-wells. Murine lung epithelial cells (MLE-15) were obtained from ATCC.
Authentication	The morphology and polarization properties of mIEC-1 and mIEC-G7 cell lines indicate their epithelial origin. The morphology and sensitivity to IFN-λ indicate the epithelial origin of MLE-15 cells.
Mycoplasma contamination	mIEC-1 and MLE-15 cell lines were tested to be mycoplasma-free when received from Tobias May laboratory or ATCC.
Commonly misidentified lines (See ICLAC register)	No commonly misidentified lines were used.

Animals and other organisms

Policy information about studies involving animals; ARRIVE guidelines recommended for reporting animal research

Laboratory animals	6-8 week old wild-type CD45.1, or congenic CD45.1 mice, Ifnar1-/-, Ifnlr1-/-, Ifnar1-/-Ifnlr1-/- mice, and Mx2 luciferase reporter mice of both genders, all on C57BL/6 background, were used for experiments, except experiments with bone marrow chimera, where older mice were used due to the time required for reconstitution of the hematopoietic compartment. All animals were bred and kept under specific pathogen-free conditions at the same location. Mice were housed in individually ventilated cages with Lab Grade Aspen Shavings Mice and kept on a standard 12 h light-dark cycle at 20-22°C and 30-70% humidity with access to food and autoclaved reverse osmosis water ad libitum.
Wild animals	The study did not involve wild animals
Field-collected samples	The study did not involve field-collected samples
Ethics oversight	All animal experiments were conducted in accordance with Rutgers University (Newark) IACUC-approved protocols.

Note that full information on the approval of the study protocol must also be provided in the manuscript.

Flow Cytometry

Plots

Confirm that:

- [x] The axis labels state the marker and fluorochrome used (e.g. CD4-FITC).
- [x] The axis scales are clearly visible. Include numbers along axes only for bottom left plot of group (a 'group' is an analysis of identical markers).
- [x] All plots are contour plots with outliers or pseudocolor plots.
- [x] A numerical value for number of cells or percentage (with statistics) is provided.

Methodology

Sample preparation	Large intestine samples were removed, open longitudinally, and gently washed with ice cold PBS. Tissue was cut into small pieces and washed twice in pre-digestion solution (1X HBSS, 5mM EDTA, 1mM DTT) for 20 min at 37°C with gentle shaking. Tissue was then enzymatically digested in collagenase D (0.05 g/100 mL PBS). Collected fractions were pooled within sample, lysed to remove red blood cells and stained.
Instrument	BD LSRFortessa X-20
Software	FACSDiva versio 8.0 for data collection and FlowJo<br>version<br>for analyses
Cell population abundance	Relative abundance of colon immune cell infiltrates on day 8 of DSS treatment is shown in Figure 3A.
Gating strategy	To enumerate colon immune cell infiltrates, cells were initially gated on live, CD45+ cells, then further evaluated using the gating strategy shown in supplementary Figure 7.

☒ Tick this box to confirm that a figure exemplifying the gating strategy is provided in the Supplementary Information.
