# Supplementary material for: Critical role of interferons in gastrointestinal injury repair
Source: Nat Commun. 2021 May 11;12:2624. doi: 10.1038/s41467-021-22928-0 (PMC8113246; doi:10.1038/s41467-021-22928-0)
Supplement: Supplementary file 4 — Description of Additional Supplementary Files [file 41467_2021_22928_MOESM4_ESM.pdf]

Description of Additional Supplementary Files

Supplementary Data 1. Protein-encoding genes up- or down-regulated above two fold (Fold Change (FC)>2 or FC<-2) in intestinal epithelial cells of 8 day old mice In response to the 3 day treatment with at least one of IFNs (IFN-α or IFN-λ) with their FC (IFN-treated over mock; FC>5 and FC<-5 are color coded) and Reads Per Kilobase of transcript per Million mapped reads (RPKM) shown as average values for three replicates. Genes encoding ribosomal proteins are not included.
